# Sex-Dependent Signatures, Time Frames and Longitudinal Fine-Tuning of the Marble Burying Test in Normal and AD-Pathological Aging Mice

**DOI:** 10.3390/biomedicines9080994

**Published:** 2021-08-11

**Authors:** Mikel Santana-Santana, José-Ramón Bayascas, Lydia Giménez-Llort

**Affiliations:** 1 Institut de Neurociències, Universitat Autònoma de Barcelona, 08193 Barcelona, Spain; mikel.santana@e-campus.uab.cat (M.S.-S.); joseramon.bayascas@uab.cat (J.-R.B.); 2 Department of Psychiatry and Forensic Medicine, School of Medicine, Universitat Autònoma de Barcelona, 08193 Barcelona, Spain; 3 Department of Biochemistry and Molecular Biology, School of Medicine, Universitat Autònoma de Barcelona, 08193 Barcelona, Spain

**Keywords:** neuroethology, behavioral neuroscience methodology, sexual differences, aging, Alzheimer’s disease, comorbidities, phobia, anxiety, OCD (obsessive-compulsive symptoms)

## Abstract

The marble burying (MB) test, a classical test based on the natural tendency of rodents to dig in diverse substrates and to bury small objects, is sensitive to some intrinsic and extrinsic factors. Here, under emerging neuroethological quantitative and qualitative analysis, the MB performance of 12-month-old male and female 3xTg-AD mice for Alzheimer’s disease and age-matched counterparts of gold-standard C57BL6 strain with normal aging unveiled sex-dependent signatures. In addition, three temporal analyses, through the (1) time course of the performance, and (2) a repeated test schedule, identified the optimal time frames and schedules to detect sex- and genotype-dependent differences. Besides, a (3) longitudinal design from 12 to 16 months of age monitored the changes in the performance with aging, worsening in AD-mice, and modulation through the repeated test. In summary, the present results allow us to conclude that (1) the marble burying test is responsive to genotype, sex, aging, and its interactions; (2) the male sex was more sensitive to showing the AD-phenotype; (3) longitudinal assessment shows a reduction in females with AD pathology; (4) burying remains stable in repeated testing; (5) the time-course of marbles burying is useful; and (6) burying behavior most likely represents perseverative and/or stereotyped-like behavior rather than anxiety-like behavior in 3xTg-AD mice.

## 1. Introduction

The behavioral and psychological symptoms associated with dementia (BPSD), including neuropsychiatric symptoms (NPS) such as anxiety and phobias, paranoia and delusion, hallucinations, stereotypes, and other disturbances, are comorbidities manifested in 50–90% of people with Alzheimer’s disease (AD) [1]. These non-cognitive problems affect their quality of life [2], are an important source of distress for patients and caregivers [3], and frequently lead to premature institutionalization [4]. Furthermore, recent studies suggest a distinct distribution of NPS comorbidities among sexes, and therefore there is a need to characterize these differences, elucidate the underlying pathophysiology, and identify better treatment targets with a gender perspective [5]. At the translational level, the modeling of BPSD/NPS in basic and preclinical research of AD under the sex perspective is also needed to develop better pharmacological and non-pharmacological preventive/therapeutical interventions that could be effectively translated into clinical scenarios. In this context, natural species-typical behaviors representing active interaction with the environment are excellent ethological scenes to reflect the interplay of cognitive and non-cognitive disturbances induced by normal and AD-pathological aging. In agreement with this, we have proven the validity of the 3xTg-AD mice to assess the impact of the disease on naturally occurring executive functions and daily life activities based on species-typical behaviors when interacting with the environment, such as burying behavior [6], and nest-building [7,8].

Burying behavior is commonly measured with the marble burying (MB) test [9], a classical behavioral test employed in rodents that exploit the tendency of these animals to dig in diverse substrates and to bury small objects, such as glass marbles, in a test cage with beddings [10]. Initially, this test was pharmacologically validated for its use to measure anxiety-related behaviors and screening for anxiolytic pharmacological drugs [11,12]. However, controversy exists regarding its specificity as it is also proposed as modeling meaningless repetitive and perseverative behaviors mimicking psychotic and obsessive-compulsive (OCD) symptoms [13]. Actually, some authors consider that for MB to be regarded as a reliable screening test for a specific assessment of a neuropsychological construct, the introduction of methodological changes or better experimental designs is needed [13,14,15]. Thus, two-zone configuration, repeated trials, and limitations inherent to MB score and ceiling/floor effects are among the experimental considerations discussed. Additionally, emerging neuroethological analysis of behavior, which integrates the sequence of behavioral events in an ethogram, may provide a better understanding of the functional role, its modulation, and underlying mechanisms than classical behavioral analysis. 

Marble burying behavior is altered in the 3xTg-AD mice. Specifically, it is enhanced in 12-month-old 3xTg-AD male mice, an age mimicking advanced stages of the disease [16,17], can be reversed by risperidone, and be modulated by handling [6,18]. In addition, we have recently proven that at 15-months of age, just 2–3 months of naturalistic isolation, which occurs when congeners die, is enough to exacerbate this behavior despite social lives since they were born, modeling the worsening of OCD described in the current COVID-19 scenario [19]. However, there are still various unresolved questions regarding the effect of sex and age factors on marble burying behavior in normal and AD-pathological aging. First, as in the case in other fields with rodent experimentation [20], the inclusion of female mice in MB testing is not the most common choice [21]. The inclusion of females in animal studies of AD is relevant, even if similar incidence between sexes is found, since risk factors may differentially affect multiple pathways and evolve into different manifestations of NPS and comorbidities [5]. Second, how aging and AD-pathological processes affect burying behavior and the MB profile evolves in a long-term perspective. This is a significant concern due to the intrinsic nature of AD, in which cognitive and psychiatric symptoms are present in early stages and worsen over time as the disease progresses [22]. Hence, to increase the translational value of experimental designs, rather than a transversal comparison of the performance at different age stages, longitudinal studies allow monitoring oof the progression of cognitive and non-cognitive deficits through an AD-pathological life-span. 

Therefore, the present study aimed to explore further the contribution of sex and aging in the normal and AD-pathological brain in marble burying behavior. We used middle-aged 3xTg-AD mice through a longitudinal study including methodological modifications (two-zone configuration, repeated trials, and time-course counting of marbles buried) to have a better approach to the possible neuropsychiatric constructs involved in their alteration, and we compared them with those presented in their non-transgenic (NTg) counterparts with the gold-standard C57BL/6 strain genetic background.

## 2. Materials and Methods

### 2.1. Animals

A total number of forty-six 12-month-old male and female mice, homozygous 3xTg-AD (males *n* = 15, females *n* = 8) and non-transgenic (NTg, males *n* = 10, females *n* = 13) mice on a C57BL/6 background (after embryonic transfer and backcrossing at least 10 generations), established in the Universitat Autònoma de Barcelona [23] were used. The 3xTg-AD mice harboring transgenes were genetically engineered at the University of California Irvine, as previously described [16]. Animals were maintained in groups of 3–4 mice per cage (Macrolon, 35 × 35 × 25 cm) filled with 5 cm thick layer of clean woodchips that were the same used for behavioral testing (Ecopure, Chips6, DateSand, UK; Uniform cross-cut wood granules with 2.8–1.0 mm chip size) and nesting materials (Kleenex, Art: 08834060, 21 × 20 cm, White). All animals were maintained under standard laboratory conditions of food and water ad libitum, 22 ± 2°C, 12 h light: dark cycle with lights on at 8:00 a.m., and relative humidity 50–60%. 

### 2.2. Experimental Design 

**Figure 1:**
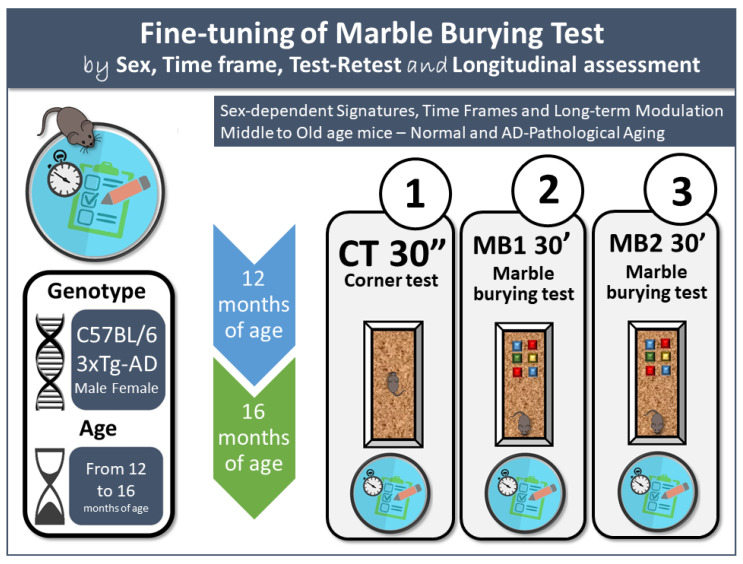
Fine-tuning of the marble burying test by sex, time frame, test–re-test, and longitudinal assessment. Experimental design: a 3-day battery of behavioral tests consisting of a corner test on day 1, a marble burying test on day 2 (MB1), and a repeated test on day 3 (MB2).

As illustred in Figure 1, animals were behaviorally assessed at middle-age (12 months of age) and re-tested four months later when they reached old age (16 months of age). In the AD-genotype, these time points correspond to two different advanced stages of the disease with the progressive development of βA and tau pathologies [17]. 

### 2.3. Behavioral Assessments

Behavioral assessments in the corner test and marble burying test under dim white light (20 lx) were conducted during the light phase of the light: dark cycle (from 10 a.m. to 1 p.m.). The tests were performed in a counterbalanced manner, by direct observation by a trained observer, blind to the genotype, and a camera’s support. All procedures were in accordance with the Spanish legislation on the “Protection of Animals Used for Experimental and Other Scientific Purposes” and the EU Directive (2010/63/UE) on this subject. The protocol CEEAH 3588/DMAH 9452 was approved the 8th of March 2019 by the Departament de Medi Ambient i Habitatge, Generalitat de Catalunya. The study complies with the ARRIVE guidelines developed by the NC3Rs and aims to reduce the number of animals used [24]. 

Day 1—Corner test (CT) was used to evaluate neophobia. The animal was placed in the center of a clean standard home cage filled with woodchip shave bedding and observed for 30 s. We measured the numbers of corners visited (CTc), the latency to perform the first rearing (CTlatR), and the number of rearings (CTr). The ratio of the number of visited corners and rearings variables (Ratio CTc/r) was calculated.

Days 2 and 3—Marble burying test (MB1) and re-test (MB2): Nine ceramic marbles were put in a standard home cage (Macrolon, 35 × 35 × 25 cm^3^) with a 5 cm thick layer of clean woodchips. The marbles were placed evenly spaced (three rows of three) in one-quarter of the cage and allowing the mice to avoid interaction with the marbles. Then, the mouse was introduced in the zone without marbles facing the wall and left to interact with the cage freely. A picture of the cage was taken every 5 min to assess the buried marbles’ progress. After 30 min the mice were gently removed from the cage, and the buried marbles were counted. Marbles were counted as buried when their surface was covered at least 90% with bedding material. The number of marbles buried was transformed in a percentage (MBx.y; x, day, y, time of measurement) for further statistical analysis. Twenty-four hours later, animals repeated the test under the same conditions.

### 2.4. Statistics

Statistical analyses were performed using SPSS 23.0 software. In the corner test, the variables recorded were analyzed by a split-plot design with the factors genotype (G), aging (A), sex (S), according to the experimental design G(2)×A(2)×S(2). ANOVA split-plot designs analyzed the number of marbles buried with the factors time (T), genotype (G), aging (A), sex (S), and day (D), according to the experimental design T(7)×G(2)×S(2)×A(2)×D(2). Post-hoc comparisons were run with Bonferroni corrections. Both the F and the degrees of freedom values were reported when it was possible. Spearman correlations were made to analyze behavioral correlates between the CT and the MB. Correlation coefficients (r) are indicated. A *p*-value < 0.05 was considered as statistically significant. Graphics were made with GraphPad Prism 6. Abbreviation: sexAgeMBday·minute (i.e, m12MB2.30, male at 12 months of age, re-test, 30 min).

## 3. Results

### 3.1. Corner Test for Neophobia

**Figure 2:**
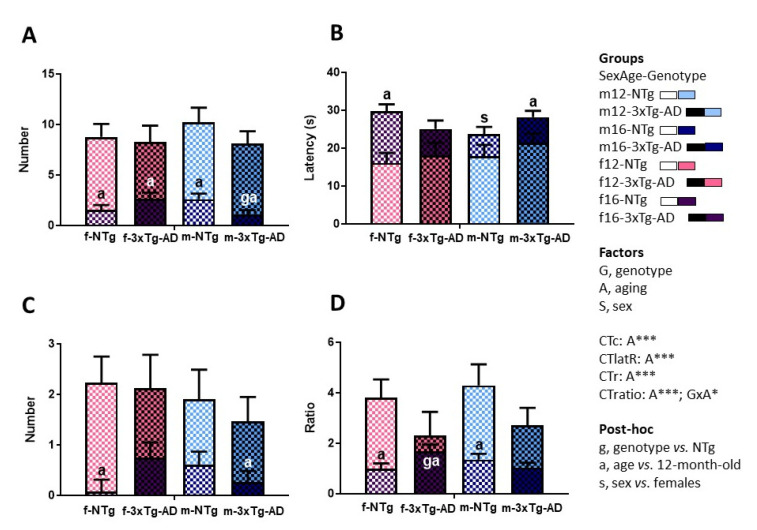
Sex and age effects in the longitudinal assessment in the corner test in mice with normal and AD-pathological aging. (**A**) Number of crossings (CTc); (**B**) Latency of rearing (CTlatR); (**C**) Number of rearings (CTr); (**D**) Ratio CTc/CTr (CTratio); Factorial analysis: G, genotype (NTg, 3xTg-AD mice); A, aging (12, 16 months of age); S, sex (male, female). * *p* < 0.05; *** *p* < 0.001.

In the corner test (Figure 2), all the variables were sensitive to the aging factor, as the longitudinal analysis showed the reduction of the number of crossings (A, F_(1,42)_ = 80.104; *p* < 0.001), the number of rearings (A, F_(1,42)_ = 24.564; *p* < 0.001), the crossings/rearings ratio (A, F_(1,42)_ = 23.903; *p* < 0.001), and, conversely, the enhancement of the latency of rearing (A, F_(1,42)_ = 18.085; *p* < 0.001). Moreover, the crossings/rearings ratio was also sensitive to the genotype and aging interaction (G×A, F_(1,42)_ = 18.085; *p* < 0.001). 

The post-hoc analysis indicated meaningful differences as described hereinafter (see g, s, a at each variable in Figure 2). Thus, at 16 months, AD-males and AD-females exhibited a lower number of crossings (g, F_(1, 42)_ = 4.474; *p* = 0.040) and higher crossings/rearings ratio (g, F_(1, 42)_ = 4.335; *p* = 0.043), respectively, than their NTg counterparts. Regarding sex, NTg-males showed a lower latency of rearing than NTg-females at 16 months of age. With aging, all the groups manifested a reduction in the number of crossings (NTg-females: *p* < 0.001; AD-females: *p* = 0.003; NTg-males: *p* < 0.001; AD-males: *p* < 0.001). However, the rearing behavior only was affected in NTg-females (CTlatR: *p* = 0.0001; CTr: *p* = 0.001) and AD-males (CTlatR: *p* = 0.048; CTr: *p* = 0.035). At 16 months, these mice showed both a delayed elicitation of rearing and a lower number of total rearings. Finally, a reduction with aging in the ratio of crossings/rearings was also presented in NTg-females (*p* = 0.001), AD-males (*p* = 0.021) and WT-males (*p* = 0.001). The post-hoc analysis of these results is also depicted in Table 1.

### 3.2. Longitudinal Assessment of Marble Burying Test and Repeated Test

**Figure 3:**
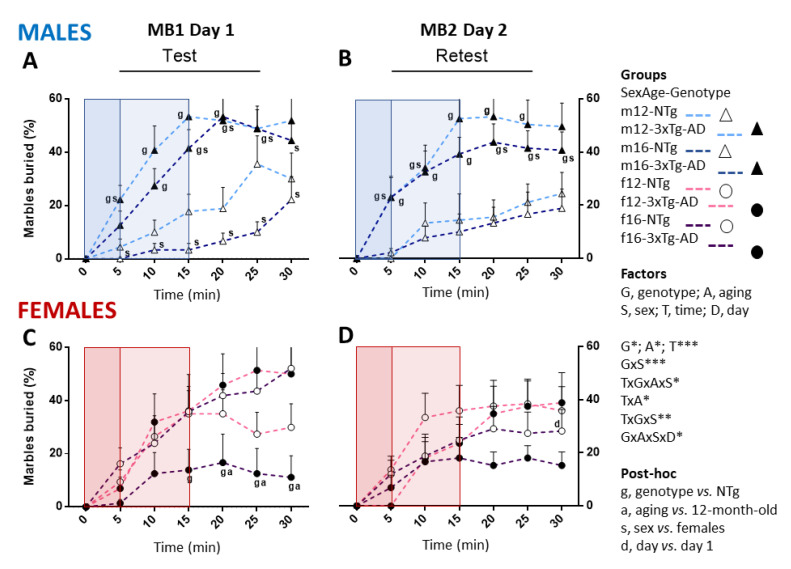
Sex, age, time, and day effects in the longitudinal assessment in the marble burying test and repeated test in mice with normal and AD-pathological aging. (**A**) Males marble burying test on day 1 (MB1 Day 1) and (**B**) re-test 24 h later (MB2 Day 2); (**C**) Females marble burying test on day 1 (MB1 Day 1) and (**D**) re-test 24 h later (MB2 Day 2); Factorial analysis: G, genotype (NTg, 3xTg-AD mice); A, aging (12, 16 months of age); S, sex (male, female); T, time (0–30 min); D, day (Day 1, Day 2). * *p* < 0.05; ** *p* < 0.01; *** *p* < 0.001.

The marble burying test (Figure 3) was sensitive to the main factors genotype (G, F_(1, 42)_ = 4.212; *p* = 0.046) and aging (A, F_(1, 42)_ = 4.325; *p* = 0.044), while sex effects depended on the genotype (G×S, F_(1, 42)_ = 12.768; *p* = 0.001). The time-course analysis indicated that time (minute) (T, F _(2.373, 99.681)_ = 68.644; *p* < 0.001) was determinant to detect genotype, sex, and age effects and interactions (T×G×A×S, F_(3.234, 135.830)_ = 3.442, *p* = 0.016; TxA, F_(3.234, 135.830)_ = 3.385; *p* = 0.017, T×G×S, F_(2.373, 99.681)_ = 5.589; *p* = 0.003), while re-test 24 h later reduced the performances of 12-month-old animals in a lower/higher intensity manner depending on the genotype and sex (G×A×S×D, F_(1,42)_ = 5.598; *p* = 0.023). In general, T×G×A×S×D interactions effects were not statistically significant (F_(3.499, 146.951)_ = 5.400; *p* = 0.061).The post-hoc analysis indicated meaningful differences as described hereinafter, providing evidence that the observation windows are critical (see g, a, s, d at each time point). The post-hoc analysis of these results is summarized in Table 2.

In the first MB testing, several meaningful differences between genotypes in the test performance were exhibited. At 12 months of age, post-hoc comparisons showed increased marble burying in AD-males compared to NTg-males (mMB5: *p* = 0.010; mMB10: *p* = 0.013; mMB15: *p* = 0.009; mMB20: *p* = 0.014), but no differences were found between females at this age. 

However, when animals reached 16 months of age, the AD-phenotype was found up-regulated in males and down-regulated in females compared to their NTg counterparts. Thus, AD-males showed increased marble burying compared to NTg-males (m16MB1·10: *p* = 0.016; m16MB1·15: *p* < 0.001; m16MB1·20: *p* < 0.001; m16MB·25: *p* = 0.001), whereas AD-females buried less marbles than their NTg counterparts (f16MB1·15: *p* = 0.046; f16MB1·20: *p* = 0.040; f16MB1·25: *p* = 0.011; f16MB1·30: *p* = 0.002). 

Besides, only the female sex exhibited longitudinal differences in the performance of the test. At 16 months of age, AD-female mice showed a lower percentage of marbles buried in the test’s final minutes (f12-16MB1·20: *p* = 0.043; f12-16MB1·25: *p* = 0.010; f12-16MB1·30: *p* = 0.010) compared to their scores at 12 months of age. 

Besides, several significant sex differences were found. At 12 months of age, significant post-hoc differences only appeared at the first five minutes of the test, where AD-males buried more marbles than AD-females (mf12MB1·5, *p* = 0.036). No differences were found between NTg mice at 12 months. Nevertheless, several meaningful differences were manifested by both AD-mice and NTg-mice at 16 months old. At this age, NTg-males buried less marbles than NTg-females early in the test (mf16MB1·5: *p* = 0.025; mf16MB1·10: *p* = 0.043; mf16MB1·15: *p* = 0.002; mf16MB1·20: *p* = 0.003; mf16MB1·25: *p* = 0.004; mf16MB1·30: *p* = 0.012). Conversely, AD-males buried a higher percentage than AD-females (mf16MB1·15: *p* = 0.011; mf16MB1·20: *p* = 0.003; mf16MB1·25: *p* = 0.003; mf16MB1·30: *p* = 0.007).

In the re-test, 24 h later, 12-month-old AD-males buried more marbles than NTg-males at several time points of the test (m12MB2·5: *p* = 0.005; m12MB2·15: *p* = 0.006; m12MB2·20: *p* = 0.007; m12MB2·25: *p* = 0.0024). Conversely, there was an absence of differences between 12-month-old AD-females and NTg-females. When the animals reached 16 months of age, genotype differences still persisted between AD and NTg males (m16MB2·5: *p* = 0.021; m16MB2·10: *p* = 0.024; m16MB2·15: *p* = 0.008; m16MB2·20: *p* = 0.004; m16MB2·25: *p* = 0.020; m16MB2·30: *p* = 0.038), but those observed between females disappeared. 

Regarding sex post-hoc differences in the re-test, they were clearly shown in the group of AD mice at both ages studied. Thus, 12-month-old AD-males buried a higher percentage of marbles than NTg-males at the beginning of the test (m12MB2·5: *p* = 0.009; m12MB2·10: *p* = 0.0045). Four months later, when they reached 16 months of age, AD-males showed very similar test and re-test patterns (m16MB2·20: *p* = 0.012; m16MB2·25: *p* = 0.0039; m16MB2·30: *p* = 0.025). However, at 16 months, differences between NTg-males and NTg-females were not found.

Finally, the percentage of marbles buried between the test and the re-test on the first and the second day of testing in all the time measures for all the groups were compared. At 12 months of age, no differences were found, while the performance of these animals at 16 months showed one difference: the NTg-females buried fewer marbles at the end time point of the second test (f16MB2·30: *p* = 0.005). Therefore, the time-course analysis is essential to unveil sex, aging, and re-test differences otherwise under-detected.

### 3.3. Corner Test and Marble Burying Test Correlations

In calculating these correlations, the variables genotype and sex were taken into account to generate the tables. The relationship between these tests was analyzed for the two ages studied. To simplify the analysis, we paid attention mainly to both the percentage of marbles buried at five and thirty minutes for the first marble test testing (MB1·5 and MB1·30, respectively). 

At 12 months of age, correlations between the CT and the MB tests were only exhibited by female mice. For NTg-female mice, the number of rearings in the CT was positively correlated with the percentage of marbles buried at both five and thirty minutes (CTr © f12MB1·5, r = 0.716; *p* = 0.006; CTr © f12MB1·30, r = 0.620; *p* = 0.024). While for AD-female mice, the number of rearings in the CT were positively correlated with the percentage of marbles buried at five minutes (CTr © f12MB1·5, r = 0.835; *p* = 0.010), the latency of rearing was inversely correlated also with the percentage of marbles buried at five minutes (CTlatR © f12MB1·5, r = −0.845; *p* = 0.008). At 16 months of age, none of the groups exhibited correlations. Among others, all these results are summarized in Table 3.

## 4. Discussion

In the present work, we corroborate the previously described higher burying of marbles in 3xTg-AD male mice at 12 months of age [6,18], and we demonstrate the complexity of factor interplay in the performance of the MB test. For the first time, we show that the higher performance in 3xT-AD male mice is also observed in the female sex, albeit statistically significant genotype differences in females are only reached at 16 months. Moreover, the longitudinal design allows the monitoring of changes in the performance with worsening of the disease only in AD-female mice, and normal aging in NTg mice, from 12 to 16 months of age, and its modulation through the repeated test. Most importantly, the time-course analysis provides a tool to discriminate the best temporal windows of observation depending on these factors.

### 4.1. New Insight of Burying Behavior in 3xTg-AD Mice

We provide evidence that the higher burying of the AD-male mice than NTg mice over 30 min [6,19] is a phenomenon sustained throughout the test and could also be scored since the beginning. For the first time, the inclusion of 3xTg-AD female mice in MB testing is reported. In contrast, despite AD-females showing higher percentages than their NTg counterparts at the end of the test, these differences did not reach statistical significance. Sexual differences were present in the AD-phenotype at the beginning of the test at 12 months old. However, with aging, these differences were exhibited in the second half of the test. In both stances, AD-female mice presented the lower percentage of marbles buried. Although it is known that the estrous cycle can affect burying activity [25,26,27], comparative studies between sexes are scarce. Those available do not find differences [28], and if they do, they are dependent on the menstrual cycle [28]. Therefore, this finding represents a step forward in the exploration of sex differences in burying behavior.

In addition, for the first time, the longitudinal design showed that pathological aging influenced MB performance. At 16 months of age, males did not show any significant difference in performance compared to their assessment at 12 months. In contrast, the performance of AD-females was lower at 16 months through all the tests, although statistical significance was reached from minute 20, and the performance of NTg-females was relatively similar at both ages. In normal aging, NTg-males showed significantly lower MB compared to NTg-females of the same age. Also, the genotype differences between male mice persisted at this age through the MB test. Therefore, we found a differential influence of how normal and AD-pathological aging affects MB performance. Firstly, aging has a differentiated response, whether accompanied by pathology or not, as shown by the fact that NTg animals do not undergo percentage changes. However, in addition, the aging of 3xTg-AD mice produced a sex-dependent differential response in their behavior, with significant differences between 16-month-old AD-females and AD-males in the second half of the MB test.

Several authors advocate for repeated trials as necessary for using MB as a model of neophobia/anxiety or OCD [13]. Following these recommendations, at both ages, we applied two consecutive days of MB testing. When the time-course of the test and re-test of all groups was compared, the standard pattern was the absence of differences in their performance at both ages, although NTg-females showed significantly lower performance at min 30 in the re-test performed at 16 months of age. As can be seen, these variations are not large enough to generate significant changes concerning their performance on the previous day. At 16 months of age, the lower performance of NTg-females eliminates both the genotype and sexual differences with AD-females and NTg-males, respectively. Moreover, the reduction of the performance of marbles buried in AD-females at 12 months suggests that aging differences with respect to AD-females at 16 months did not show up, and caused the temporal amplification of sexual differences at the beginning of the test regarding AD-males. For the rest, genetic and sexual differences manifested by AD-males at both ages were still conserved. Although slight variations are exhibited in some measures, they do not change their manifested behavioral phenotype interpretation.

### 4.2. Corner Test and Its Relationship with Marble Burying Test in 3xTg-AD Mice

In the present work, we did not find genotype differences in neophobia, contrary to what occurred on other occasions [6,19]. However, we did observe a reduction in mouse activity and a slowdown of latency due to aging. This occurs in all variables, although it does not always occur in all groups in a statistically significant way, although a tendency can be appreciated.

The relationship between the burrowing percentage in the MB and the CT variables could be described as inconsistent and poor. To simplify its interpretation, we focused our attention on the first measure, which should be more sensitive to neophobia, and the last one, for comparative purposes. If we hypothesize that the higher percentage of buried marbles is due to anxiety, a clear relationship should be visible between the two tests, especially for AD-males. However, correlations only appeared in both NTg and AD female mice. To summarize, these results would be in line with the poor relationship showed between the MB and other tests for assessing anxiety-like behavior in other studies [29,30,31].

### 4.3. Marble Test as a Model of Anxiety-Like or OCD-Like Behavior in 3xTg-AD Mice?

With all of the previously discussed, the modeling of anxiety-like behavior in the burying behavior of this animal model is certainly questionable. First of all, due to the utilization of the two-zone configuration, the animals can avoid the marbles, so we can assume that the interaction with the marbles is, to some extent, voluntary. Then, it would be expected that AD-mice would show passive avoidance of marbles, which is not the case. These results would be consistent with those shown by other studies using a two-zone configuration [11,14,31,32,33,34]. Furthermore, in the re-testing, no change would indicate that habituation to a stressful situation is produced, either assuming two different fight-to-flight scenarios: a case where the animals were so frightened of the marbles that they buried as a defensive strategy (AD-mice), or they would avoid their interaction with them (NTg mice). However, they interact as well, but to a lesser extent. Moreover, although there are no clear genotype differences in neophobia, measured through CT, differences still appeared in burying behavior in MB. Finally, correlations between the burying percentages and the CT variables are scarce and inconsistent. A possible hypothesis to support the anxiety-like modeling of burying behavior could be that the inherited anxiety trait of these mice could make their response to marbles resistant to habituation [35,36] and thereby invoking either active burying or passive avoidance behavior as coping strategies [36,37,38,39,40,41]. Since 3xTg-AD mice present higher baseline anxiety [23], which produces differentiated anxious responses depending on the test [18], the previous hypothesis is still possible. Interestingly, we already reported that other animal models for anxiety, such as the A1 receptor knock out mice, also show reduced habituation [42]. Moreover, in our precedent Gimenez-Llort and Alveal-Mellado’s work [19], 3xTg-AD mice showed a higher freezing behavior in the open-field test accompanied with higher amounts of marble burying, contrary to NTg-mice. Therefore, although the modeling of anxious-like behavior is questionable, with the methodology employed it is not completely discardable.

It seems pretty clear that regardless of whether they bury more or fewer marbles, their performance in this test is persistent and stable over time, in concordance with other studies with repeated MB application [28,31,32,43,44,45]. This event would support the current practice of using burying behavior as an indication of OCD-like behavior, although this approach also has certain validity concerns [13,45,46]. While OCD may be a risk factor for developing AD [47,48], it is unlikely to be the construct modeled in the 3xTg-AD mice. Due to the repetitive and perseverative nature of burying activity, this behavior could represent NPS such as perseverative behavior and/or stereotyped behavior. Both are NPS usually present in patients with Alzheimer’s and other dementias [49,50,51]. In addition, 3xTg-AD mice have been shown to present more significant errors due to preserverative behavioral hopelessness paradigms [52] and attentional tasks [53], and greater presence of stereotyped behaviors at early stages of the disease [23]. Therefore, it is quite possible that burying behavior in 3xTg-AD mice reflects perseverative and/or stereotyped-like behavior rather than anxiety-like behavior. However, this is not necessarily denying that anxiety may influence the performance on the marble test, mainly through differentiated coping strategies.

Far from discussions about which pathology models the test in our animal model, what remains clear is that the burying behavior is stable and resistant to repetition, regardless of the group or whether the animals show high or low percentages. Therefore another way of interpreting the results would be to look at them with a neuroethological perspective, in which burying is an inherent behavior of the animal [13], in this case, sensitive to AD-pathology and with differentiated response depending on sex, aging, and their interactions. Considering that burying represents the application of digging [13], defined as the displacement of a substrate mainly using forepaws [54], to a more complex task, it would be expected that digging was the sensitive behavior to the AD pathology. Thus, 3xTg-AD mice should show a similar profile in other tests involving digging behavior shown in the MB. Therefore, further research is needed to confirm this hypothesis.

### 4.4. Integrating Our New Findings into Our Previous Knowledge of The Burying Behavior of 3xTg-AD Mice

The inter-test stability in the burying behavior also has important implications for supporting the conclusions developed in previous works of our research group with the 3xTg-AD mice. In Torres-Lista’s previous studies [6,18], chronic administration of risperidone reduced the number of marbles buried compared to their pre-treatment testing for both AD-male and NTg-male mice. Moreover, this number was modulated in saline groups, albeit presenting a different pattern depending on the genotype. In AD-mice, as happened with risperidone, the number of marbles buried was reduced, whereas for NTg-males, this number was higher. These changes in the marble activity were attributed to an effect of the repetitive handling for the administration of the saline compounds. The results obtained in the present study would support that conclusion since, without the handling, the animal’s burying behavior should have been unaltered in the resetting. We attributed the reduction of marble activity in the AD saline group to the anxiolytic effect of handling [55,56,57], and the increase in marble activity in NTg-males with an increase in their emotional state. However, this claim remains unclear partly for our present results and partly because burying behavior can be enhanced in mice by stressing the animal through non-pharmacological intervention [58,59,60]. Thus, it may have been possible that the repetitive subcutaneous administration of the saline compound acted as a stressor affecting the inherited burying behavior of each group and thereby either increasing or decreasing it depending on their inherited pattern.

### 4.5. Benefits of Implementing the Time-Course of Marble Buried

This is the first time that time-course of marbles buried by 3xTg-AD mice in MB was studied as a methodological novelty. As we previously explained in the method, it consisted of counting the number of marbles buried at five minute intervals until the end of the test. Although we have already commented on their results in the previous section, we would like to make some remarks to promote its use. First of all, its application is easy and affordable. It can be done through photography or video, not interfering in the normal development of the test. Moreover, the intervals can be easily adapted to the needs of the study, although it would be advisable to include at least one measurement in the middle of the test. The reason for this is that, at least in our model, differences usually appear at 10–15 min, and the score at that time does not differ significantly from the score obtained at 30 min (analysis not shown). To us, the most important reason for its use is that it gives us valuable information about the behavioral pattern of the animal throughout the test, helping us to establish a more accurate profile of the animal and the possible differences between them. This may be especially important in pharmacological and non-pharmacological interventions as well, as such interventions could modify the pattern of the animal and not just the final test score. Concerning the latter, time-course could save many nightmares to researchers using the MB. If we consider only the final measurement, we could erroneously conclude that there are significant differences (false positive) or not (false negative) between the two groups when in fact, throughout the test, this was not the case. In our experiment, we can see an example of each. As a false positive in the final measurement, we have that the 16-month-old AD-females burying percentage is significantly different regarding their score on the previous testing, but only in that score. While as a false negative, we see that in the last measurement of the re-test, the significant difference between AD-males and NTg-males at 12 months disappears when there have been significant differences in all the previous scores. In addition, although the counting of marbles through the test has existed for a long time [43], its use is not widespread. It is difficult to find examples of its use in the literature, although they certainly exist [59,61]. As in our experiment, the differences found at the end of the test are usually manifested from the first measurements and are relatively stable over the time course. Considering the foregoing, we consider that the advantages of its application far outweigh the costs of its implementation.

### 4.6. Methodological Limitations of the Study

Although, in our view, the data support the above discussions, certain methodological limitations may influence the degree of certainty of our conclusions. Here, we’ll discuss them to warn the reader regarding the possible impact of these limitations on interpretability and, consequently, the conclusions drawn.

First, it is necessary to discuss the statistical analysis employed. MB is commonly analyzed using linear models, such as the *t*-test and ANOVA. In our case, we have also employed a split-plot ANOVA. However, as Lazic [15] points out, this type of analysis may be inadequate because, as the number of marbles is a counted data, it does not meet the requirements of this type of analysis, leading to 95% confidence intervals that include impossible values (less than zero or greater than the number of marbles present), misleading p-values, and impossible predictions. There are other more appropriate (non-parametric) types of analysis. However, in our case, we decided to use the analysis for several reasons: 1) to be in line with our previous studies, 2) to use the most commonly used method in the literature, and 3) the complexity of the experimental design made it very difficult to use non-parametric analysis techniques.

On the other hand, there are several limitations in ascertaining which construct the MB test relates to or models. In the case of anxiety, there are more classical tests than the CT for measuring anxiety-like behavior (e.g., open field, elevated plus maze). We used the CT because in our previous studies [6], there were correlations concerning the MB. However, as mentioned above, other studies have explored the relationship between anxiety tests and the MB and obtained relatively poor results [29,30,31], so it is unlikely that this would be any different in our case. In perseverative behavior, we also do not have an alternative behavior (e.g., grooming) in the MB or another test to validate this hypothesis. However, in this case, the design relies on the resistance to habituation of the marble test, as is the case in other experimental tests e.g., [31]. The main intention of this paper is not to conclude what behavior models the MB in the 3xTg-AD model, although we do hypothesize, based on the data obtained, that it could be due to the previous points of the discussion.

All in all, it is clear that more research is needed to explore these questions further and overcome the limitations present in this study.

## 5. Conclusions

In summary, the present results allow us to conclude that (1) the marble test is responsive to genotype, sex, aging, and its interactions; (2) the male sex was more sensitive to showing the AD-phenotype; (3) longitudinal study shows a reduction of burying in females with AD pathology; (4) burying remains stable in repeated testing; (5) the time-course of marbles buried is a useful methodological modification; and (6) burying behavior in the MB test most likely represents perseverative and/or stereotyped-like behavior rather than anxiety-like behavior in 3xTg-AD mice. More research is needed in the 3xTg-AD mice to approach further the modeling of perseverative and stereotyped-like behavior in MB and to be able to verify if the profile shown in the MB test is transferable to other tests that imply digging behavior.

## Figures and Tables

**Table 1:** Post-hoc analysis of genotype, sex, and aging differences in the corner test.

Post-Hoc Analysis CORNER TEST	Genotype (vs. NTg Mice)		Sex (vs. Female Mice)		Aging (vs. 12-Month-Old Mice)	
Behavioral variable	12 mo	16 mo	12 mo	16 mo	Each group	
CTc	All n.s.	Males *p* = 0.040	All n.s.	All n.s.	fNTg f3xTg-AD mNTg m3xTg-AD	*p* < 0.001 *p* = 0.003 *p* < 0.001 *p* < 0.001
CTlatR	All n.s.	All n.s.	All n.s.	NTg *p* = 0.024	fNTg f3xTg-AD mNTg m3xTg-AD	*p* < 0.001 n.s. n.s. *p* = 0.048
CTr	All n.s.	All n.s.	All n.s.	All n.s.	fNTg f3xTg-AD mNTg m3xTg-AD	*p* = 0.001 n.s. n.s. *p* = 0.035
CTratio	All n.s.	Females *p* = 0.043	All n.s.	All n.s.	fNTg f3xTg-AD mNTg m3xTgAD	*p* = 0.001 n.s. *p* = 0.001 *p* = 0.021

Abbreviations: mo, months-old; CTc, number of crossings; CTlatR, latency of rearing; CTr, number of rearings; CTratio, CTc/CTr ratio; fNTg, female NTg mice; f3xTg-AD, female 3xTg-AD mice; mNTg, male NTg mice; m3xTg-AD, male 3xTg-AD mice; n.s., *p*-value value is not statistically significant.

**Table 2:** Post-hoc analysis of genotype, sex, and aging differences in the marble test.

Post-Hoc Analysis MARBLE TEST	Genotype (vs. NTg Mice)		Sex (vs. Female Mice)		Aging (vs. 12 mo)	Repeated Test(vs. Day 1)
Behavioral variable	12 mo	16 mo	12 mo	16 mo	Each group	Each group
Day 1 (MB1)						
MB1.5	Females *p* = 0.010	All n.s.	3xTg-AD *p* = 0.036	NTg *p* = 0.025	All n.s.	All n.s.
MB1.10	Females *p* = 0.013	Males *p* = 0.016	All n.s.	NTg *p *= 0.043	All n.s.	All n.s.
MB1.15	Females *p* = 0.009	Females *p* = 0.046 Males *p* = 0.000	All n.s.	NTg *p* = 0.002 3xTg-AD *p* = 0.011	All n.s.	All n.s.
MB1.20	Females *p* = 0.014	Females p = 0.040 Males *p* = 0.000	All n.s.	NTg *p* = 0.003 3xTg-AD *p* = 0.003	f3xTg-AD *p* = 0.043	All n.s.
MB1.25	All n.s.	Females *p* = 0.011 Males *p* = 0.001	All n.s.	NTg *p* = 0.004 3xTg-AD *p* = 0.003	f3xTg-AD *p* = 0.010	All n.s.
MB1.30	All n.s.	Females *p* = 0.002	All n.s.	NTg *p* = 0.012 3xTg-AD *p* = 0.007	f3xTg-AD *p* = 0.010	All n.s.
Day 2 (MB2)						
MB2.5	Males *p* = 0.005	Males *p* = 0.021	3xTg-AD *p* = 0.009	All n.s.	All n.s.	All n.s.
MB2.10	All n.s.	Males *p* = 0.024	3xTg-AD *p* = 0.045	All n.s.	All n.s.	All n.s.
MB2.15	Males *p* = 0.006	Males *p* = 0.008	All n.s.	All n.s.	All n.s.	All n.s.
MB2.20	Males *p* = 0.007	Males *p* = 0.004	All n.s.	3xTg-AD *p* = 0.012	All n.s.	All n.s.
MB2.25	Males *p* = 0.024	Males *p* = 0.020	Alln.s.	3xTg-AD *p* = 0.039	All n.s.	All n.s.
MB2.30	All n.s.	Males *p* = 0.038	All n.s.	3xTg-AD *p* = 0.025	All n.s.	fNTg *p* = 0.005

Abbreviations: mo, months-old; (MBx.y), MB, Marble test; x, day; y, time (accumulated counts); fNTg, females NTg; n.s., *p*-value value is not statistically significant.

**Table 3:** Corner test and marble burying test Spearman correlation analysis.

		CTc	CTlatR	CTr	CTratio
fNTg (*n* = 13) at 12 moa					
MB1.5	Spearman correlation Sig. (2-tailed)	n.s.	n.s.	0.716 ** 0.006	n.s.
MB1.10	Spearman correlation Sig. (2-tailed)	n.s.	n.s.	0.617 * 0.025	n.s.
MB1.15	Spearman correlation Sig. (2-tailed)	n.s.	n.s.	0.697 ** 0.008	n.s.
MB1.20	Spearman correlation Sig. (2-tailed)	n.s.	n.s.	0.631 * 0.021	n.s.
MB1.25	Spearman correlation Sig. (2-tailed)	n.s.	n.s.	n.s.	n.s.
MB1.30	Spearman correlation Sig. (2-tailed)	n.s.	n.s.	0.620 * 0.024	n.s.
fNTg at 16 moa					
MB1.25	Spearman correlation Sig. (2-tailed)	0.568 * 0.043	n.s.	n.s.	n.s.
f3xTg-AD (*n* = 8) at 12 moa					
MB1.5	Spearman correlation Sig. (2-tailed)	n.s.	−0.845 ** 0.008	0.0835 ** 0.010	n.s.
All the other groups					
MB1.all	Spearman correlation Sig. (2-tailed)	n.s.	n.s.	n.s.	n.s.

Only statistically significant correlations are indicated. Abbreviations: fNTg, females NTg; f3xTg-AD, females 3xTg-AD mice; moa, months of age; (MBx.y) MB, marble test; x, day; y, time (accumulated counts); fNTg Sig., significant; **, correlation significant at the 0.01 level (2-tailed), *, correlation significant at the 0.05 level (2-tailed).

## Data Availability

Not applicable.
